# *Morus alba* Leaf Lectin (MLL) Sensitizes MCF-7 Cells to Anoikis by Inhibiting Fibronectin Mediated Integrin-FAK Signaling through Ras and Activation of P^38^ MAPK

**DOI:** 10.3389/fphar.2017.00034

**Published:** 2017-02-07

**Authors:** Jayaram Saranya, Ganesan Shilpa, Kozhiparambil G. Raghu, Sulochana Priya

**Affiliations:** ^1^Agro-Processing and Technology Division, CSIR-National Institute for Interdisciplinary Science and TechnologyThiruvananthapuram, India; ^2^Academy of Scientific and Innovative ResearchNew Delhi, India

**Keywords:** *Morus alba*, anoikis, fibronectin, integrin, focal adhesion kinase

## Abstract

Lectins are a unique class of carbohydrate binding proteins/glycoproteins, and many of them possess anticancer properties. They can induce cell cycle arrest and apoptosis, inhibit protein synthesis, telomerase activity and angiogenesis in cancer cells. In the present study, we have demonstrated the effect of *Morus alba* leaf lectin (MLL) on anoikis induction in MCF-7 cells. Anoikis induction in cancer cells has a significant role in preventing early stage metastasis. MLL treatment in monolayers of MCF-7 cells caused significant detachment of cells in a time and concentration dependent manner. The detached cells failed to re-adhere and grew even to culture plates coated with different matrix proteins. DNA fragmentation, membrane integrity studies, annexin V staining, caspase 9 activation and upregulation of Bax/Bad confirmed that the detached cells underwent apoptosis. Upregulation of matrix metalloproteinase 9 (MMP-9) caused a decrease in fibronectin (FN) production which facilitated the cells to detach by blocking the FN mediated downstream signaling. On treatment with MLL, we have observed downregulation of integrin expression, decreased phosphorylation of focal adhesion kinase (FAK), loss in FAK-integrin interaction and active Ras. MLL treatment downregulated the levels of phosphorylated Akt and PI3K. Also, we have studied the effect of MLL on two stress activated protein kinases p38 MAPK and JNK. p38 MAPK activation was found to be elevated, but there was no change in the level of JNK. Thus our study substantiated the possible antimetastatic effect of MLL by inducing anoikis in MCF-7 cells by activation of caspase 9 and proapoptotic Bax/Bad by blockage of FN mediated integrin/FAK signaling and partly by activation of p38 MAPK.

## Introduction

Lectins are carbohydrate binding proteins/glycoproteins with diverse biological applications and are considered as one of the potent anticancer agents in future. The anti tumor activities of lectins from different sources are well established with the proper illustration of its mechanism of action (Fang et al., 2011; Huang et al., 2012). A heterodimeric glycoprotein isolated from *Abrus precatorius* mediated caspase induced cell death *in vitro* and *in vivo* in human liver cancer cells. It decreased Akt phosphorylation, HSP 90, CD 31 and Ki67 expression in HepG2 xenografted nude mice (Mukhopadhyay et al., 2014). Lectin from the fungus *Sclerotium rolfsii* exerted cytotoxic effects in human colon cancer cells by altering the expression of the genes involved in apoptosis, cell cycle regulation, MAPK and JNK signaling cascades (Barkeer et al., 2015). Mulberry belongs to the family of plants called *Moraceae* contains a number of lectins with varying sugar specificity. We have reported previously that an *N*-acetyl galactosamine lectin from *Morus alba* showed cell cycle arrest and caspase dependent apoptosis in human colon and breast cancer cells (Deepa and Priya, 2012; Deepa et al., 2012).

Interaction of cells with the neighboring cells as well as to the extracellular matrix (ECM) maintains the normal development and homeostasis. Anoikis is a type of programmed cell death triggered by the loss of proper cell-ECM interaction. The ability of cancer cells to evade from the programmed cell death once it detach from the primary tumor microenvironment (anoikis resistance) helps the cells to survive in the circulatory system for a long time which causes metastasis. Induction of anoikis in detached cancer cells is an efficient way to prevent the reoccurrence of cancer in distant organs (Simpson et al., 2008; Westhoff and Fulda, 2009). Breakdown of anoikis leads to the occurrence of cancer in epithelial as well as non-epithelial cells (Shanmugathasan and Jothy, 2000; Hu et al., 2001). Complex regulatory mechanisms are involved in the induction of anoikis and its resistance in cancer cells. Anoikis can be either through the intrinsic pathway by the activation of mitochondrial proapoptotic class 2/3 BCl2 family proteins or through extrinsic death receptor mediated activation of caspase 8. Once the cells detached from the ECM, Bax-Bak oligomers assemble on the mitochondrial outer membrane; thus the Bim and Bid are getting activated. When the cell-ECM contact is lost, association of Bim with the dynein complex ends and it move to mitochondria. Moreover, phosphorylation of Bim by ERK and PI3K/Akt targets this protein for proteasomal degradation (Chiarugi and Giannoni, 2008). Transcriptional regulation of Noxa and Puma, the class 3 BCl2 family of proteins by p53 have major significance in fibroblast anoikis (Nakano and Vousden, 2001). In the extrinsic pathway overexpression of the negative form of death receptor FADD failed to recruit caspase 8 to DISC complex and inhibit anoikis (Rytomaa et al., 1999).

Integrins and cadherins, the proteins involved in the cell-ECM and cell–cell communication have an important role in regulating anoikis. The ligated conformation of integrin with FAK stimulates the downstream signaling promoting cell proliferation through PI3K/Akt pathway which causes anoikis resistance whereas its unligated form activates anoikis. Interaction of cadherin-catenin complex with actin filaments allowing the cell–cell adhesion and communication through PI3K/Akt or Raf/ERK pathways also regulate anoikis (Frisch and Screaton, 2001; Malagobadan and Nagoor, 2015). The active PI3K-Akt pathway in normal proliferating cells inhibit the mitochondrial translocation of activated Bax, thus preventing the cells from undergoing apoptosis (Tsuruta et al., 2002). Activated Akt has multiple targets of action in the cell death signaling cascade like inactivation of caspase 9, phosphorylation and proteasomal degradation of Bad, activation of NF- kappa B and inhibition of Forkhead transcription factors in sensitizing anoikis resistance (Chiarugi and Giannoni, 2008). Additionally, oncogenic Ras protein facilitates the cancer cells in ECM detached condition and block anoikis (Mason et al., 2016). The mitogen activated protein kinase p38 have a dual role in cancer. It induces upregulation of several proapoptotic genes in many human tumors, whereas situations are also there it enhances the cell proliferation and drug resistance (Cuenda and Rousseau, 2007; Wagner and Nebreda, 2009).

Compounds with anoikis inducing activity have significant implications in cancer therapeutics because it prevents cancer metastasis. Lupalbigenin, the isoflavone from the medicinal plant *Derris scandens* prevented the anchorage independent growth of lung cancer cells by downregulating the phosphorylation of Akt and ERK proteins (Ausawasamrit et al., 2015). Geraniin, the hydrolyzable tannins, suppresses the invasion and anoikis resistance in lung cancer cells by inhibiting transforming growth factor β (Ko, 2015). The cytotoxic effects of the compounds from *M. alba* in various cancer cells have been reported previously (Naowaratwattana et al., 2010; Deepa et al., 2013). The flavonoid morin and the isoflavone morusin from *M. alba* induced apoptosis in human pancreatic and colon cancer cells by modulating BCl2 family of proteins, Fas receptors and STAT 3 signaling (Hyun et al., 2015; Kim et al., 2016). In this study, we have explored the mechanism of induction of anoikis by a previously reported cytotoxic lectin from *M. alba*. (Deepa and Priya, 2012).

## Materials and Methods

### Cell Line and Reagents

Human breast cancer cells, MCF-7 was purchased from NCCS (Pune, India). 3-(4,5-dimethylthiazol-2-yl)-2,5-diphenyltetrazolium bromide (MTT), Col I, FN, LN, dimethyl sulfoxide (DMSO), 4′, 6-diamidino-2-phenylindole dihydrochloride (DAPI), annexin V staining kit, acridine orange, ethidium bromide, RIPA buffer, protein A beads and agarose were purchased from Sigma Chemical, Inc. (St. Louis, MO, USA). Dulbecco’s modified Eagle medium (DMEM), Fetal bovine serum (FBS), trypsin and antibiotic-antimycotic (100X) solution were obtained from Himedia Laboratories (Mumbai, India). Caspase 9 assay kit and mitochondria fractionation kit were purchased from Biovision (Milpitas, CA, USA). Anchorage independent plate (polyHEMA coated) was purchased from Cell Biolabs (San Diego, CA, USA). Protein estimation kit (Bradford method) was purchased from Thermo Scientific (Rockford, IL, USA). Primary antibodies against Bax, Bad, FAK, pFAK, α5 integrin, and HRP conjugated anti mouse and anti rabbit secondary antibodies were from Santa Cruz Biotechnology (Santa Cruz, CA, USA). Ras activation assay kit, and antibodies against MMP-9, MMP-2, FN, Col I, LN, Akt, pAkt, PI3K, pPI3K, JNK, pJNK, P^38^MAPK, pP^38^MAPK, actin and GAPDH were bought from Cell Signaling Technology (Beverly, CA, USA). Chemiluminescence western blot developing kit was from Bio-Rad (Hercules, CA, USA). All other reagents used in the study were of analytical grade or high quality available in the market.

### Purification of MLL

*Morus alba* leaf lectin was purified from *M. alba* leaves by ammonium sulfate fractionation followed by immobilized metal ion affinity chromatography and anion exchange chromatography on DEAE [23]. The activity of MLL was measured using hemagglutination assay and the purity of the lectin was analyzed by silver stained SDS-PAGE (results are given as **Supplementary Figure S1**).

### Cell Culture and Cytotoxicity Studies

Breast cancer (MCF-7) cells were maintained in DMEM supplemented with 10% FBS, 100 U/ml of antibiotic/antimycotic solution and incubated at 37°C in a humidified 5% CO_2_ atmosphere. At 70% confluency, the cells were subcultured and the doubling time was found to be ∼30 h. After subculturing the cells were reseeded in 96 well culture plates and treated with varying concentrations of MLL (2, 5, and 10 μg/ml) for different time intervals (4, 8, 12, 24, and 48 h). MTT assay was done to measure the cytotoxicity and the results were expressed as the percentage of viable cells by measuring the absorbance at 560 nm of the dissolved formazan crystals produced under each condition (Ahmad et al., 2012). Also we have confirmed the anoikis induction caused by MLL by seeding equal number (2 × 10^4^) of cells (control as well as MLL treated) on anchorage independent plate first for 24 h and then transfered to normal plate for 4 h to check the attachment. Cell viability was checked by MTT assay.

### Phase Contrast Imaging for Observing the Detachment of Cells

Morphological analysis as well as the detachment of cells at different time points, under control and MLL treated conditions were done using the phase contrast microscope Nikon Eclipse TS-100 (Nikon Instruments Inc., Melville, NY, USA). The detached cells (after 24 h treatment) were reseeded in culture dishes coated with different matrix proteins (Col I, FN, and LN) and observed under the microscope for re-adherence and growth. Matrix proteins were coated on the culture dishes at a concentration of 50 μg/ml before seeding the cells and polylysine coated plates served as the control.

### Apoptosis Analysis

Nuclear fragmentation assay was done using agarose gel electrophoresis and DAPI staining. Intact and fragmented DNA from control and detached cells (after 24 h treatment) were isolated using apoptotic ladder kit (G-Biosciences, USA) and electrophoresed on agarose gel incorporated with ethidium bromide and visualized using gel doc (Bio-Rad, Hercules, CA, USA).

4′, 6-diamidino-2-phenylindole dihydrochloride staining was used to study the nuclear fragmentation within the cells (Tanious et al., 1992). Control as well as the detached cells (after 24 h treatment) were stained with DAPI (300 nM) and subjected to fluorescent imaging (BD Pathway^TM^ Bioimager system, USA).

Acridine orange/Ethidium bromide staining was used to check the membrane integrity during apoptosis (Cohen, 1993). 25 μl of cell suspension from control and detached cells after 24 h treatment (1 × 10^4^ cells/ml) was mixed with 1 μl acridine orange/ethidium bromide solution (one part each of 100 μg/ml of acridine orange and 100 μg/ml of ethidium bromide in PBS) for 5 min. Cells were observed and images were taken using fluorescent microscope (BD Pathway^TM^ Bioimager system, USA).

Annexin V staining was done to detect the number of live, necrotic and apoptotic cells as per the instructions from the manufacturer (Sigma Chemical, Inc. St. Louis, MO, USA). The detached cells (after 24 h treatment) along with control were treated with 50 μl of double label staining solution (AnnCy3 and 6CFDA). After 10 min incubation, washed with PBS and examined the cells under fluorescent microscope (BD Pathway^TM^ Bioimager system, USA).

### Caspase 9 Activity Assay

Measurement of the Caspase 9 activity in the cell lysates of control and detached cells (after 24 h treatment) was done using fluorimetric assay kit by following the instructions from the manufacturer (Biovision). The assay was based on measuring the absorption maxima of the cleaved product AFC (7-amino-4-trifluoromethyl coumarin) at 505 nm from the substrate LEHD-AFC on caspase 9 action. Results were expressed as relative units in terms of fluorescence.

### Gelatin Zymography

Matrix metalloproteinase activity was studied using gelatin zymography in cell culture supernatants from control and 24 h MLL treated cells (Kiran et al., 2006). The medium was subjected to gelatin impregnated polyacrylamide gel electrophoresis. After electrophoresis, the gel was washed with 2.5% Triton X-100 solution and incubated in 50 mM Tris–HCl containing 5 mM CaCl_2_, 0.2 M NaCl at pH 7.5 for 24 h. The gel was stained with Coomassie brilliant blue (0.25%) followed by destaining. White bands of enzyme digested regions were visualized against a dark blue background.

### Ras Activation Assay

Measurement of the active form of Ras was done using assay kit by following the instructions from the manufacturer (Cell Signaling Technology). It helped to measure the activation of Ras-GTPase in the cell by immunoprecipitating the active GTP bound Ras with glutathione resin and then determined by western blot analysis using the monoclonal antibody against Ras.

### Enzyme Linked Immunosorbent Assay (ELISA)

Effect of MLL on matrix proteins production was measured using ELISA (Engvall and Perlman, 1971). The medium was collected from control as well as the cells treated with MLL (5 and 10 μg/ml) for 24 h. ELISA was carried out using specific primary antibody (1:500 dilution) and HRP conjugated secondary antibody (1:1000 dilution) and the color was developed using *o*-dianisidine as the substrate. The concentrations of the antigens were estimated by measuring the absorbance of the colored HRP product spectrophotometrically at 490 nm in a microplate reader (Biotek Synergy 4, Winooski, VT, USA). The same protocol was used for determining the activity of Bax in the mitochondrial fraction of control as well as detached cells using anti Bax antibody.

### Co-immunoprecipitation

Co-immunoprecipitation was done to study the interaction of integrin and FAK under normal as well as the 24 h MLL treated condition. Briefly, 200 μl of the cell extracts were incubated overnight at 4°C with primary antibody against α5 integrin. Then protein A agarose beads were added (20 μl of 50% bead slurry) and incubated with mild rocking for 3 h at 4°C. The tubes were centrifuged for 30 s at 4°C and washed the pellet five times with 500 μl of 1X cell lysis buffer. The pellets were resuspended in 20 μl 2X SDS sample buffer, mixed well and spun for 30 s. The samples were heated to 95°C for 5 min and centrifuged for 1 min at 14,000 × *g*. The input, immunoprecipitate and flow through fractions were loaded on SDS-PAGE gel and were analyzed by western blotting using anti-FAK and anti-integrin antibodies.

### Preparation of Cell Extracts and Western Blot Analysis

Whole cell extracts were prepared in RIPA buffer containing protease inhibitor cocktail. Estimation of total protein was done using Bradford method. Mitochondrial and cytoplasmic fractions were separated by using standard kit by following the manufacturer’s instructions. An equivalent amount of protein (∼40 μg/well) from control as well as treated cells (24 h) were electrophoresed in SDS-PAGE and transferred to nitrocellulose membrane. The membrane was blocked with 5% skim milk and probed with respective primary antibodies. After washing, HRP conjugated secondary antibodies were added and incubated. Protein bands were developed by chemiluminescent method. Images of interested proteins bands were captured and analyzed using ChemiDoc MP^TM^ System with Image Lab^TM^ Software, Bio-Rad (Hercules, CA, USA).

### Statistical Analysis

Minimum three independent experiments were performed for all assays. Results were expressed as mean ± SD of three experiments and data were analyzed using one way ANOVA using SPSS statistical analysis program. *p*-values of < 0.05 were considered as statistically significant.

## Results

### MLL Induced Anoikis in MCF-7 Cells

**FIGURE 1:**
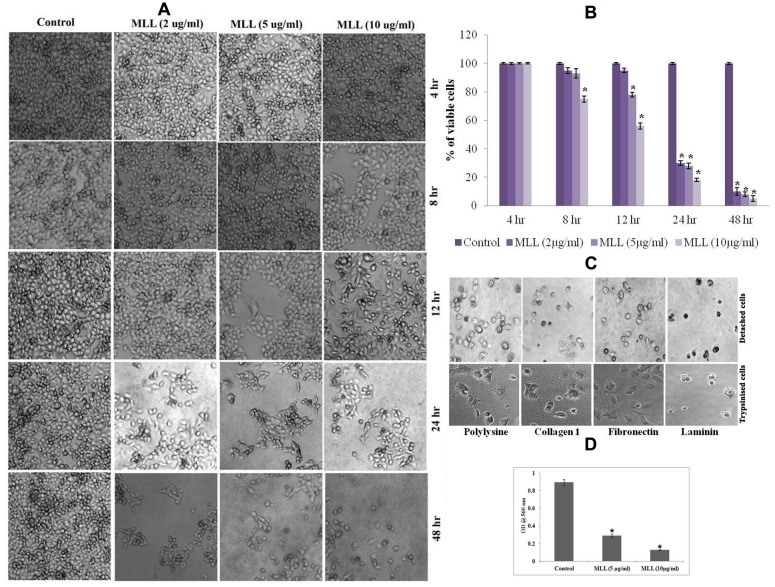
**(A)** Phase contrast image showing the detachment of cells under control/*Morus alba* leaf lectin (MLL) treated conditions in a time and concentration dependent manner. Magnification -4×. **(B)** MTT assay indicating the percentage of viable cells under control and varying concentrations of MLL for specific time intervals. Values are average of three independent experiments ± SD and ^∗^*p* < 0.05 when compared to control. **(C)** Phase contrast image showing detached cells on cell culture plates coated different matrix proteins. The cells were not able to attach and grow further. But the trypsinised cells (control) seeded on matrix proteins were attached and grow further. Magnification -10×. **(D)** MTT assay showing the growth of cells after anoikis induction by MLL. Values are average of three independent experiments ± SD and ^∗^*p* < 0.05 when compared to control.

In order to study the effect of MLL on detachment induced cell death in MCF-7 cells, confluent monolayers of cells were treated with varying concentrations (2, 5, and 10 μg/ml) of MLL for different time intervals (4, 8, 12, 24, and 48 h) and observed for detachment of cells from the culture dishes. Morphological observations (**Figure 1A**) indicated that the detachment of cells started during 8 h of incubation with 10 μg/ml MLL treatment. During 12 h, the detachment started with 5 μg/ml as well similar to that of 10 μg/ml MLL treatment. During 24 and 48 h, significant number of cells was detached even at 2 μg/ml concentration. Also, we have observed the percentage of viable cells remained in the plate after MLL treatment by MTT assay. More than 70% of cells were detached from the plates during 24 and 48 h in all the concentrations of MLL treatment (**Figure 1B**). We have also studied whether these detached cells can re-adhere and proliferate when sufficient conditions are given. For this, the detached cells were collected and reseeded on culture plates coated with different matrix proteins (Col I, FN, and LN and polylysine coated plates served as the control). The plates were kept under standard culture condition for 24 h and observed under microscope. After 24 h also the cells were floating and not able to adhere and grow even if they were provided with different matrix support for attachment. We have seeded the trypsinised cells (from the control plate) in different matrix coated plates as experimental control which got adhere to the plate. The results are given in **Figure 1C**. Anoikis induction by MLL was also confirmed by first seeding the cells (equal number of cells with and without MLL) on anchorage independent plate for first 24 h and subsequently in normal plate for 4 h. Here the control cells were able to attach and re-grow in the normal plate, but the cells treated with MLL could not. The cell viability in the normal plates were measured by MTT assay and the results are given in **Figure 1D**. So the results indicated that the MLL caused the detachment of cells from the plate and failed to re-adhere and grow further.

**FIGURE 2:**
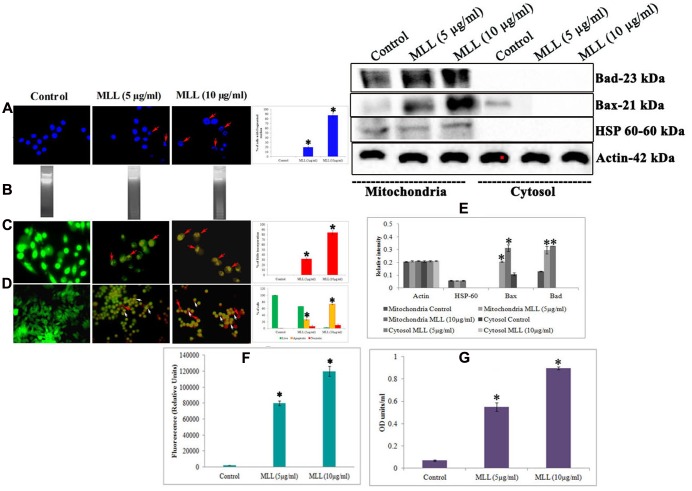
**(A)** DAPI staining showing the nuclear fragmentation. Control cells possess well defined nuclear morphology whereas the detached cells under MLL treated conditions showed significant DNA fragmentation (indicated by red arrows). Magnification -40×. Bar diagram showing the percentage of fragmented DNA. Values are average of three independent experiments ± SD and ^∗^*p* < 0.05 when compared to control. **(B)** Agarose gel electrophoresis showing the nuclear fragmentation of control and MLL treated detached cells. **(C)** Acridine orange/Ethidium bromide staining indicating the disruption in the membrane integrity in the detached cells (indicated by red arrows). Control cells exhibited green fluorescence because of the intact cell membrane. Magnification -40×. Bar diagram showing the percentage of EtBr fluorescence. Values are average of three independent experiments ± SD and ^∗^*p* < 0.05 when compared to control. **(D)** Annexin V staining showing the live (green fluorescent cells), necrotic (red fluorescent cells indicated by red arrows) and apoptotic cells (orange fluorescent cells indicated by white arrows) in control as well as MLL treated detached cells. Magnification -40×. Bar diagram showing the quantitative measurement. Values are average of three independent experiments ± SD and ^∗^*p* < 0.05 when compared to control. **(E)** Western blot analysis indicating the upregulation of Bax and Bad in the mitochondrial fractions in MLL treated detached cells. HSP-60 was used as the mitochondrial marker protein and actin served as the internal control. **(F)** Effect of MLL on the activity of caspase 9 of control and MLL treated detached cells. Values are average of three independent experiments ± SD and ^∗^*p* < 0.05 when compared to control. **(G)** Effect of MLL on the activity of Bax in the mitochondrial fraction of control and MLL treated detached cells. Values are average of three independent experiments ± SD and ^∗^*p* < 0.05 when compared to control.

Further to this, we have done experiments (DNA fragmentation, membrane integrity measurement, phosphatidyl serine translocation, activity of caspase 9, Bax and Bad) to confirm the programmed death in the detached cells. Significant fragmentation of the nuclear DNA was observed in DAPI staining (**Figure 2A**) as well as agarose gel electrophoresis (**Figure 2B**) in detached cells compared to control. The percentage of cells with fragmented nucleus was significantly increased in a concentration dependent manner in the detached cells compared to control cells (shown as bar diagram). The integrity of the cell membrane was checked by acridine orange/ethidium bromide staining. Because of the loss of membrane integrity in apoptotic cells, the detached cells appeared as orange (colocalization of ethidium bromide along with acridine orange) compared to control cells where the cells gave bright green fluorescence (only acridine orange has taken up by the cells). The percentage of EtBr incorporation was measured and plotted as a bar diagram which indicated that the MLL treated cells, the membrane damage was significant. The results are given in **Figure 2C**. Phosphatidyl serine translocation to the outer membrane is an indication of apoptosis. Quantitative fluorescence staining using annexin V in control as well as detached cells gave the percentage of live (green fluorescent), apoptotic (orange fluorescent), and necrotic cells (red fluorescent). The results in **Figure 2D** indicated that the apoptotic cells were significantly more in detached cells on treatment with MLL. Bax and Bad are two proapoptotic BCl2 family protein often getting activated during apoptosis. Mitochondrial translocation of Bax from cytosol and oligomerisation of Bad in the mitochondrial membrane are responsible for the intrinsic apoptotic pathway. Western blot analysis (**Figure 2E**) showed an upregulation of both these proteins in detached cells compared to control in the mitochondrial fractions. Bar diagram indicated the relative intensity of each band in western blot analysis. Here we have used HSP-60 as the mitochondrial marker protein to show the change in expression was in mitochondria. The activity of caspase 9, the major caspase involved in the intrinsic apoptotic pathway was also upregulated in the detached cells (**Figure 2F**). We have also studied the activity of Bax in the mitochondrial fractions separated from control as well as detached cells by indirect ELISA. The results given in **Figure 2G** showed the upregulation of active Bax in the mitochondria in the detached cells which is also a positive indication of apoptosis. These results indicated that MLL promoted detachment induced cell death (anoikis) in MCF-7 cells in a time and concentration dependent manner.

### MLL Altered the Level of ECM Protein and ECM Degrading Enzyme

**FIGURE 3:**
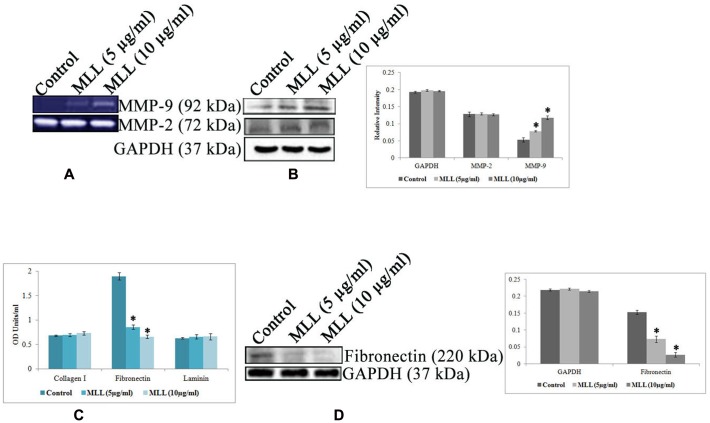
**(A)** Gelatin zymography showing the upregulation of MMP-9 under MLL treated conditions. MMP-2 activity remains constant in control as well as MLL treated conditions. **(B)** Western blot analysis of MMP-9 and MMP-2. MMP-9 expression was found to be elevated significantly under MLL treatment compared to control. Bar diagram showing the quantitative measurement. Values are average of three independent experiments ± SD and ^∗^*p* < 0.05 when compared to control. **(C)** Indirect ELISA showing the expression of various matrix proteins under control and MLL treated conditions. Increased level of FN expression was downregulated on treatment with MLL. Values are average of three independent experiments ± SD and ^∗^*p* < 0.05 when compared to control. **(D)** Western blot analysis showing the downregulation of FN under MLL treated conditions. GAPDH served as the internal control. Bar diagram showing the quantitative measurement. Values are average of three independent experiments ± SD and ^∗^*p* < 0.05 when compared to control.

Anoikis is a type of cell death induced by improper cell-ECM contacts and in this process, ECM proteins and ECM degrading enzymes have a major role to play. We have analyzed the effect of MLL on the production of two major matrix degrading enzymes MMP-2 and MMP-9 by gelatin zymography (**Figure 3A**) and western blot analysis (**Figure 3B**). The results indicated that there was an upregulation of MMP-9 activity in MLL treated condition compared to control, whereas the activity of MMP-2 was unaltered in the same experimental conditions. Also, we have checked the production of three important matrix proteins Col I, FN, and LN in control and MLL treated conditions using indirect ELISA. The level of Col I and LN was minimal and unaltered in control as well as MLL treated cells. The level of FN was significantly high in control cells and the level was reduced to more than 50% upon treatment with MLL (**Figure 3C**). The downregulation in the production in FN was also confirmed by western blot analysis (**Figure 3D**).

### MLL Inhibited Integrin Mediated Downstream Signaling through FAK

**FIGURE 4:**
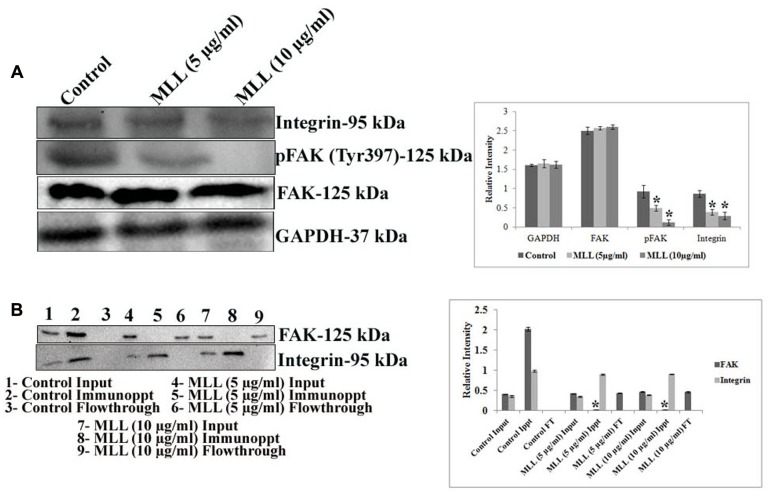
**(A)** Western blot analysis showing the decreased level of expression of integrin and pFAK under MLL treated conditions compared to control. FAK level remained unaltered. GAPDH was used as the internal control. A quantitative level of expression of different proteins is given in the bar diagram. Values are average of three independent experiments ± SD and ^∗^*p* < 0.05 when compared to control. **(B)** Western blot analysis using FAK and integrin antibody after co-immunoprecipitation of FAK and integrin from the control and MLL treated cell lysates using anti integrin antibody. The intense band in IP when blotted with FAK in the control indicated the interaction between FAK and integrin. On treatment with MLL, integrin-FAK interaction lost and no bands were detected in the IP region (detected in the flow through). The equally intense bands in the IP regions of control and treated cells when blotted with integrin antibody indicated that the integrin levels in the cell lysates taken for IP were same. A quantitative level of expression of different proteins is given in the bar diagram. Values are average of three independent experiments ± SD and ^∗^*p* < 0.05 when compared to FAK of control immunoprecipitate.

The downstream signaling mediates the interaction of cells with ECM through the transmembrane receptor protein integrin. Since we have found that the level of FN was significantly reduced upon MLL treatment, we were interested in studying the level of integrin which interacts with FN. Since the α5 subunit of integrin is involved in the FN mediated signaling, we have studied its level using western blot analysis. The results in **Figure 4A** indicated a downregulation of α5 integrin upon treatment with MLL compared to control. The next effector molecule in this signaling cascade is the FAK and the phosphorylation of FAK achieves further signaling. We have studied the level of FAK and its phosphorylated form by western blot analysis. The results (**Figure 4A**) indicated that there was a significant downregulation in the phosphorylation of FAK in MLL treated cells compared to control. We have studied the interaction between FAK and integrin by co-immunoprecipitation. First immunoprecipitation was done in the cell lysates (from control, MLL 5 μg/ml and MLL 10 μg/ml) with integrin antibody and the input, immunoprecipitate and flow through fractions were electrophoresed and immunoblotted with FAK and integrin antibody. In this experiment in control IP, we got a band when blotted with FAK indicated the association between FAK and integrin. The IP bands were stronger compared to input band indicating that the IP worked. But in the treated cell lysates, FAK bands were absent in IP but were detected in the flow through indicating the FAK-integrin interactions were not there. When blotted with integrin antibody, intense bands (compared to input) of equal intensity were observed in the IP of control as well as treated conditions. Nothing we could detect in flow through. These results are given in **Figure 4B**. Second immunoprecipitation was done with a control antibody (other than integrin, but from same species) and repeated the western blot using FAK and integrin antibody. Here in the immunoprecipitate could not detect any band but were detected in the flow through. This indicted that the IP band came in the previous experiment in the control was due to the interaction between FAK and integrin and not because of the binding to the FC binding sites of the antibody used. These experiment data we have included as a **Supplementary Figure S2** in the manuscript.

So the decreased level of α5 integrin, reduced pFAK level and diminished interaction between integrin and FAK indicated that MLL inhibited the integrin mediated downstream signaling through FAK.

### MLL Downregulated the Active Ras in MCF-7 Cells

**FIGURE 5:**
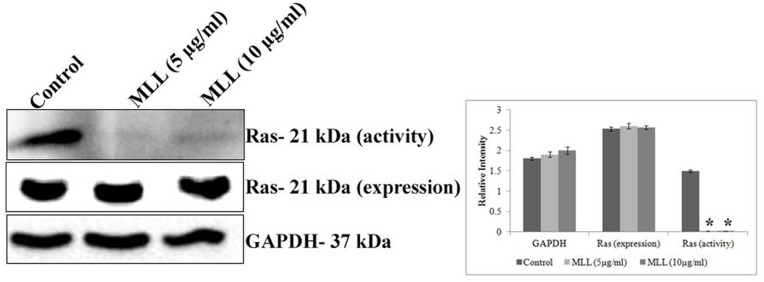
**Western blot analysis showing the activity and protein expression levels of Ras in control and MLL treated cells.** There was significant downregulation of activity of Ras but the protein expression remains the same. GAPDH was used as the internal control. Bar diagram showing the quantitative measurement. Values are average of three independent experiments ± SD and ^∗^*p* < 0.05 when compared to the Ras activity of control.

Ras superfamily of proteins belong to the class small GTPase are involved in switching on the genes that induce cell growth and proliferation and overexpression of Ras activity leads to cancer. The increased FAK signaling may be mediated through increased Ras activity. In this context, we have studied the effect of MLL on Ras activity (active GTP bound Ras) and Ras expression (western blot analysis of total cell lysate using anti ras antibody) in MCF-7 cells. The results in **Figure 5** indicated a downregulation in the activity of Ras, upon treatment with MLL when compared to control cells but the protein expression remains the same.

### MLL Inhibited the Expression of pPI3K, pAkt and Upregulated pP^38^ MAPK

**FIGURE 6:**
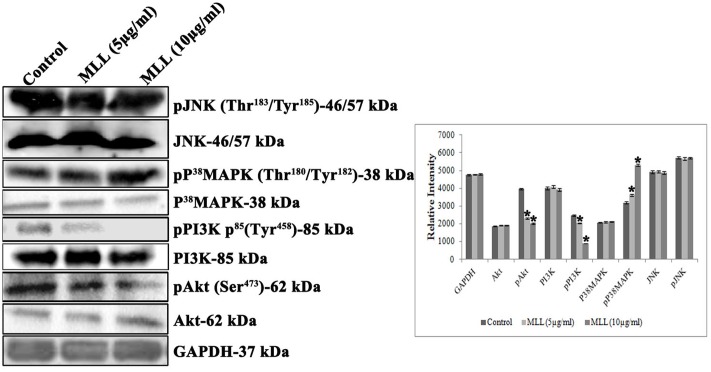
**Western blot analysis showing the levels of Akt, pAkt, PI3K, pPI3K, P^38^MAPK, pP^38^MAPK, JNK, and pJNK under control and MLL treated conditions.** Downregulation in the expression of pPI3K, pAkt and upregulation of pp38 MAPK was noticed on treatment with MLL compared to control. A quantitative level of expression of different proteins is given in the bar diagram. Values are average of three independent experiments ± SD and ^∗^*p* < 0.05 when compared to control.

PI3Ks are the heterodimeric lipid kinases involved in cell survival, growth, adhesion, transformation and apoptosis. It is the next effector molecule in the FAK signaling cascade and phosphorylated activation of PI3K resulted in the phosphorylation of Akt resulted in cell growth by preventing apoptosis. We have studied the expression of PI3K, pPI3K, Akt, and pAkt in MCF-7 cells treated with MLL. Significant reduction in the expression of pPI3K and pAkt was found when treated with MLL compared to control (**Figure 6**). p38 MAPK and JNK are belonging to the mitogen activated protein kinases induced by stress stimuli and have the significant role in inducing apoptosis. We have studied the effect of MLL on these two protein kinases and the results indicated that there was an upregulation in the phosphorylation of p38 MAPK, but no change in the expression of JNK/pJNK (**Figure 6**).

## Discussion

Interaction of tumor cells with the microenvironment is a regulating factor in its motility, invasiveness and metastatic potential. The initiation of programmed cell death can be in many ways. The process of anoikis starts with deregulation in the interaction of cells with ECM, converge to caspase activation and finally cell death through the interplay between the main molecular pathways in cell death mechanism. Sensitization of anoikis in cancer cells has significant implications for controlling the metastatic process. Water extracts of *Andrographis paniculata* down-regulated the metastatic gene (TM4SF3) expression in esophageal cancer cells (Yue et al., 2015). Apigenin, the trihydroxy flavone, inhibited the melanoma cell migration by inducing anoikis by minimizing the integrin level and phosphorylation of FAK and ERK (Hasnat et al., 2015). Moscatilin, a dibenzyl derivative present in orchid sensitizing anoikis in human lung cancer cells by decreasing the level of Akt and ERK, expression of caveolin 1 and anti apoptotic MCl-1 protein (Busaranon et al., 2016). The endogenous lectin, galectin1 promoted anoikis in cancer cells by inhibiting the signaling through α5β1 integrin, the FN receptor present on the cell surface (Sanchez-Ruderisch et al., 2011). The anticancer activity of lectins from various sources was studied previously (Panda et al., 2014; Xiao et al., 2015; Singh et al., 2016). But anoikis inducing effects of lectin purified from any natural sources are seldom reported. In the present study, we have showed that MLL induced anoikis in MCF-7 cells in a time and concentration dependent manner. Also, we have found that the mediation of anoikis is through the intrinsic pathway of apoptosis as evidenced by upregulation of caspase 9, Bax and Bad. Following detachment from the ECM, Bax-Bak oligomerizes at the mitochondrial outer membrane which interacts with mitochondrial channel proteins such as voltage dependent ion channels, allowing membrane permeabilization and release of cytochrome c. The formation of apoptosome with cytochrome c, Apaf1 and inactive caspase 9 leads to activation of caspase 9 with subsequent activation of terminal caspases and execution of cell death (Chiarugi and Giannoni, 2008).

The matrix protein FN and the MMP-9 have definite role in cancer cell proliferation and invasion. In normal proliferating cells, FN helps to maintain cell adhesion with ECM through its interaction with α5β1 integrin. To detach the cells from the ECM meshwork, termination of this FN mediated downstream signaling and activation of enzymes that degrade the ECM should happen. Once analyzed the major matrix proteins, we found that the level of FN was significantly come down, and there was upregulation of MMP-9 upon treatment with MLL. In many metastatic conditions, upregulation of MMP-2 and 9 was reported (Li et al., 2015; Xu et al., 2015; Ma et al., 2016). But in the present study, upregulation of MMP-9 helped to detach from the adherent condition before it underwent cell death. Also we found that the detached cells are undergoing apoptosis, there won’t be any further proliferation at distant organs. In this context, we have further examined the downstream signaling mediated by FN. FN interact with the cell surface receptor α5β1 integrin and phosphorylate FAK for signal transduction. The key proteins involved in integrin mediated signaling are FAK, integrin linked kinase (ILK), Src tyrosine kinase, PI3K, ERK and the adapter protein Shc. MLL treatment caused a decrease in α5 integrin level, FAK phosphorylation, and integrin-FAK interaction in MCF-7 cells. Integration of integrin with FAK cause phosphorylation of its tyrosine residue and suppression of this interrupt the invasion and metastasis of cancer cells. Activated FAK can transduce signals to any one of the downstream targets like ERK1/2, MAPK, or PI3K-Akt (Harnois et al., 2004). FAK activation lead to adhesion and invasion of pancreatic cancer cells via ERK1/2 pathway (Sawai et al., 2005). DZ-50, the quinazoline based compound induce anoikis in renal cell carcinoma by inhibiting FAK and Akt signaling (Sakamoto et al., 2011). Increased FN expression enhances integrin signaling through FAK activation and this is interconnected with Ras activation through different signaling modes. Increased FN triggers phosphorylation of FAK at Tyr397 and Tyr925 results in binding of Src family of protein tyrosine kinase and Grb2. Grb2/Shc association lead to activation of ERK2/MAPK. Mutation studies revealed that Tyr 925 phosphorylation of FAK specifically mediate this process through activation of Ras (Schlaepfer and Hunter, 1997). FAK phosphorylation at Tyr 861 elevated H-Ras and oncogenic transformation by associating with p130CAS (Lim et al., 2004). Also elevated levels of FAK activate Shc, the upstream mediator of Ras activity by diminishing the interaction of Ras with its negative modulator p120RasGAP (Hecker et al., 2004).

Oncogenic activation of ras proteins upregulates RAF-MEK-ERK pathway to enhance the mitogenic action. Also by interacting with p110 subunit of PI3K, activated Ras mediated oncogenic transformation (Castellano and Downward, 2011). PI3K is the upstream kinase to be activated in Akt survival pathway. We have found the downregulation of activated Ras, pPI3K and phosphorylated Akt under MLL treated condition indicated the suppression of PI3K/Akt pathway in MLL induced anoikis. Inhibition of PI3K/Akt pathway by MLL helped in the mitochondrial translocation of Bax. Reports are there that dephosphorylated Akt can promote the conformational changes in the Bax protein which helped its translocation to mitochondria (Yamaguchi and Wang, 2001). The essential trace element zinc induced anoikis in lung cancer cells through downregulation of Akt pathway (Pramchu-Em et al., 2016). Increased activity of Akt can suppress the stress activated protein kinase p38 and JNK involved in apoptosis. We found that the phosphorylated level of p38 was increased upon MLL treatment whereas the JNK/pJNK level remained unaltered. Activated p38 regulate apoptosis and cell cycle arrest by the downstream activation of many protein kinases and transcription factors. p38 activation also contributes to mitochondrial cytochrome c release, mitochondrial translocation of Bax protein and extrinsic/intrinsic caspase activation. ROS mediated activation of p38 increases the level of active caspases in HeLa cells (Lenassi and Plemenitas, 2006). Rotenone and metformin induced oxidative stress mediated upregulation of p38 and apoptosis in MCF-7 cells (Deng et al., 2010; Queiroz et al., 2014). The anticancer saponin, dioscin induced apoptosis and cell cycle arrest in human laryngeal cancer by elevating the levels of p38, caspase 9 and Bax (Si et al., 2016). Anoikis induction in colon cancer cells by a cholesterol derivative anicequol involves activation of p38MAPK and a rho dependent kinase (Tanakara et al., 2013).

**FIGURE 7:**
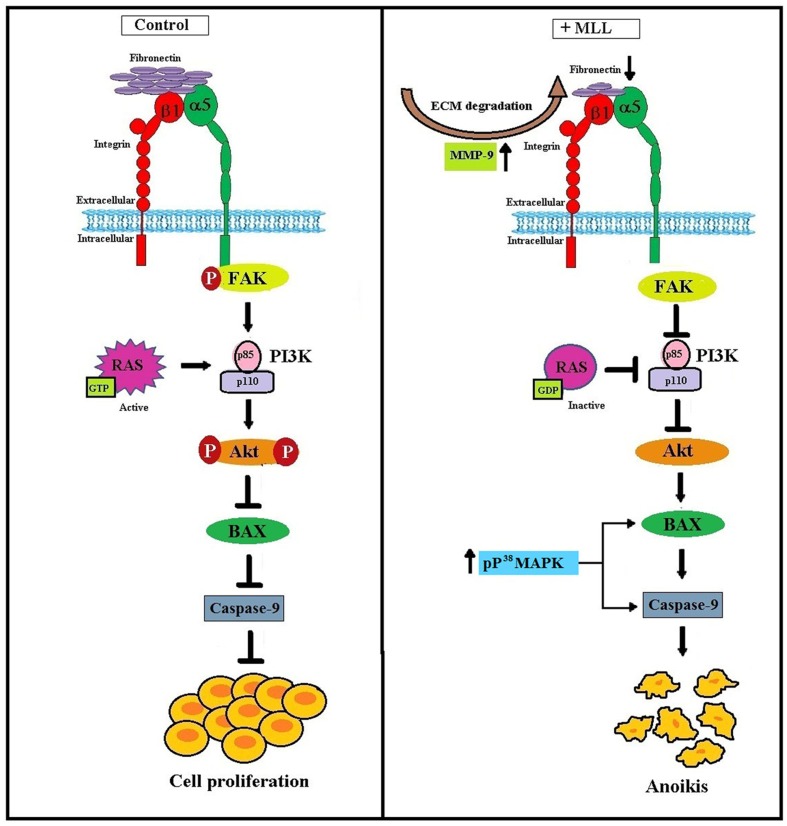
**Schematic representation of MLL induced anoikis in MCF-7 cells: Upregulation of MMP-9 as a result of MLL treatment caused ECM degradation and reduced FN expression.** Reduced FN level decreased its interaction with the integrin receptor and downstream signaling. In association with this, integrin –FAK interaction and phosphorylation FAK inhibited. This in turn decreased the expression of PI3K and phosphorylation of Akt. In the unphosphorylated condition Akt promote the expression of proapoptotic Bax and caspase 9 which caused programmed cell death (anoikis). Inactivation of Ras protein also inhibited the level of PI3K. Moreover, phosphorylated upregulation of P^38^MAPK activate Bax and caspase 9 which in turn contributed to the anoikis process.

From our results, we conclude that MLL induced anoikis in MCF-7 cells by downregulating the integrin mediated FAK signaling. Activated Ras and p38 MAPK also take part in this process for the upregulation of proapoptotic Bax and caspase 9. **Figure 7** clearly summarizes the molecular signaling pathway with which the MLL exerted its anoikis effects in MCF-7 cells.

## Author Contributions

Work design: SP. Conducted the experiments: JS and GS. Contributed in discussion and modification: KR.

## Conflict of Interest Statement

The authors declare that the research was conducted in the absence of any commercial or financial relationships that could be construed as a potential conflict of interest.

## Supplementary Material

**FIGURE S1 | Purification of MLL from *Morus alba* leaves using IMAC and DEAE.** **(A)** Purification table for MLL. **(B)** Silver stained SDS-PAGE showing the purity of MLL.

**FIGURE S2 | Western blot analysis using FAK and integrin antibody after co-immunoprecipitation of FAK and integrin from the control and MLL treated cell lysates using control antibody.** The integrin and FAK were detected only in the flow through fractions and no bands were detected in the immunoprecipitate indicating that the immunoprecipitation did not work here. A quantitative level of expression of different proteins is given in the bar diagram.

## Abbreviations

Col I: collagen I
ECM: extracellular matrix
FAK: focal adhesion kinase
FN: fibronectin
LN: laminin
MLL: *Morus alba* leaf lectin
MMP: matrix metalloproteinase
