# Supplementary figures and images for: Morus alba Leaf Lectin (MLL) Sensitizes MCF-7 Cells to Anoikis by Inhibiting Fibronectin Mediated Integrin-FAK Signaling through Ras and Activation of P38 MAPK
Source: Front Pharmacol. 2017 Feb 7;8:34. doi: 10.3389/fphar.2017.00034 (PMC5293820; doi:10.3389/fphar.2017.00034)

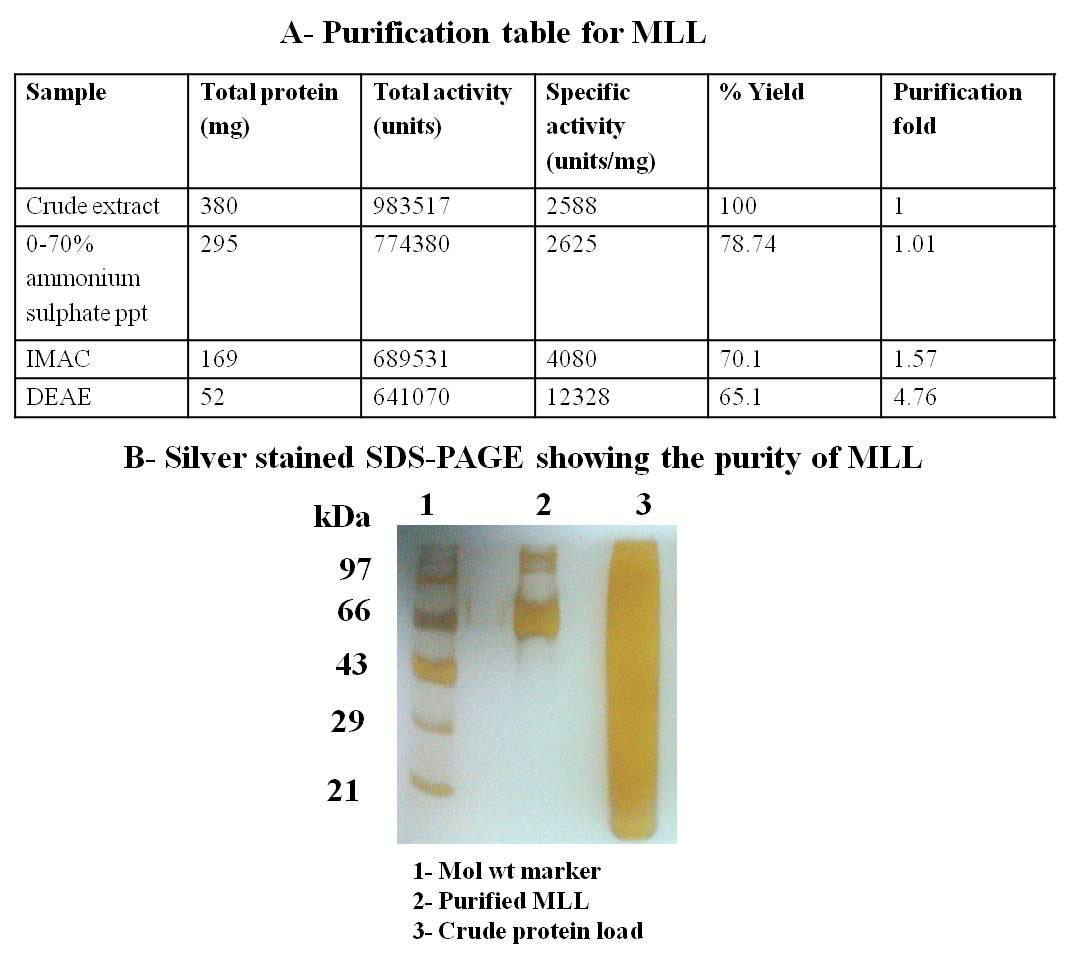

Supplement: Supplementary file 1 [file Image_1.JPEG]

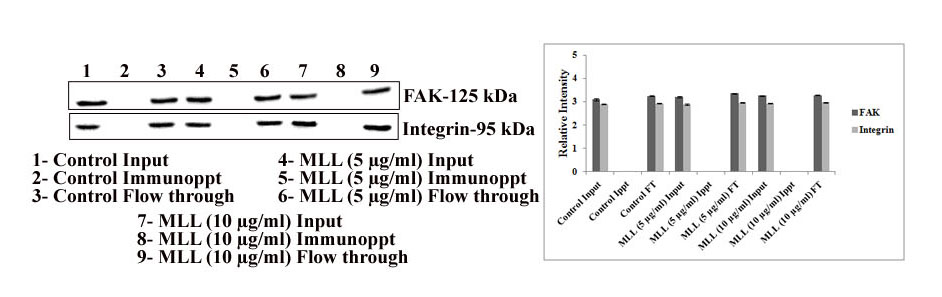

Supplement: Supplementary file 2 [file Image_2.JPEG]
